# Diadenosine pentaphosphate regulates dendrite growth and number in cultured hippocampal neurons

**DOI:** 10.1007/s11302-023-09944-z

**Published:** 2023-05-29

**Authors:** M. Diez-Zaera, A. Ruiz-Calvo, J. I. Diaz-Hernandez, A. Sebastián-Serrano, P. Aivar, B. Alvarez-Castelao, J. Pintor, M. Diaz-Hernandez, M. T. Miras-Portugal

**Affiliations:** 1 Departamento de Bioquímica y Biología Molecular, Facultad de Veterinaria, Universidad Complutense de Madrid, Avda. Puerta de Hierro S/N, 28040 Madrid, Spain; 2 Departamento Ciencia de La Salud, Facultad Ciencias Biomédicas y de La Salud, Universidad Europea de Madrid, 28670 Madrid, Spain; 3 Departamento de Bioquímica y Biología Molecular, Facultad de Óptica y Optometría, Universidad Complutense de Madrid, 28037 Madrid, Spain; 4 Instituto de Investigación Sanitaria del Hospital Clínico San Carlos, IdISSC, Madrid, Spain

**Keywords:** P2X1, Ap_5_A, Dendrite growth

## Abstract

During the establishment of neuronal circuits, axons and dendrites grow and branch to establish specific synaptic connections. This complex process is highly regulated by positive and negative extracellular cues guiding the axons and dendrites. Our group was pioneer in describing that one of these signals are the extracellular purines. We found that extracellular ATP, through its selective ionotropic P2X7 receptor (P2X7R), negatively regulates axonal growth and branching. Here, we evaluate if other purinergic compounds, such as the diadenosine pentaphosphate (Ap_5_A), may module the dynamics of dendritic or axonal growth and branching in cultured hippocampal neurons. Our results show that Ap_5_A negatively modulates the dendrite’s growth and number by inducing transient intracellular calcium increases in the dendrites’ growth cone. Interestingly, phenol red, commonly used as a pH indicator in culture media, also blocks the P2X1 receptors, avoided the negative modulation of Ap_5_A on dendrites. Subsequent pharmacological studies using a battery of selective P2X1R antagonists confirmed the involvement of this subunit. In agreement with pharmacological studies, P2X1R overexpression caused a similar reduction in dendritic length and number as that induced by Ap_5_A. This effect was reverted when neurons were co-transfected with the vector expressing the interference RNA for P2X1R. Despite small hairpin RNAs reverting the reduction in the number of dendrites caused by Ap_5_A, it did not avoid the dendritic length decrease induced by the polyphosphate, suggesting, therefore, the involvement of a heteromeric P2X receptor. Our results are indicating that Ap_5_A exerts a negative influence on dendritic growth.

## Introduction

Neurons are highly polarized cells that have structurally and functionally distinct processes called axons and dendrites. Neural connections through these structures create the neural circuits crucial to the flow of information transmission in the brain [1]. The establishment of these circuits requires the development of axons and dendrites under distinct steps including elongations, branching, pathfinding, and synaptic connection which are regulated both by intracellular and extracellular signaling cues [2–5]. Either permissive (e.g., neurotrophins) and repulsive signals (e.g., semaphorins, ephrins, netrins, slits) have been identified. These signals can be short-ranged and locally derived or secreted, diffusible, and long-ranged [3, 6]. Our group was pioneer describing extracellular ATP negatively controls axonal growth and branching in cultured hippocampal neurons [7] and inhibits neuritogenesis in Neuro 2a neuroblastoma cells [8]. Similarly, ATP was found to reduce neurite extension from motoneuron-containing neural tube explants of E12 rat embryos [9].

ATP and other adenine nucleotides behave as neurotransmitters in the central nervous system activating specific ionotropic P2X or metabotropic P2Y receptors [10]. Among these, the diadenosine polyphosphates (Ap_n_A), formed by two adenosines joined by a variable number of phosphates, have been also described as neurotransmitters, in particular Ap_4_A, Ap_5_A, and Ap_6_A in brain tissue [11]. These compounds are stored together with ATP and other neurotransmitters in synaptic vesicles being released after synaptic terminal stimulation. Although the existence of a specific receptor for Ap_n_A, tentatively named P4, was postulated [12], some of these compounds can also activate both P2X and P2Y receptors in a way similar to ATP agonists or potentiating responses triggered by ATP [13]. Indeed, Ap_n_A may activate at least four recombinant homomeric P2X receptors (P2X1–4), being the homomeric P2X1R and P2X3R those that present a higher affinity to these compounds [13, 14]. Since P4 receptor has not been cloned yet, it cannot be rule out the possibility that this receptor may be a heterodimer of two or more P2X subunits.

Interestingly, previous studies reported that activation of P2Rs promotes neurite outgrowth in different organotypic brain slice co-cultures. Although the authors did not identify the stoichiometry of the P2X receptor involved, the pharmacological and immunological studies suggested the involvement of P2X1-3 subunits [15, 16]. Later investigations revealed that P2X1R-mediated Ca^2+^ influx triggered by DA-9801, a well-standardized *Dioscorea rhizome* extract, potentiates nerve growth factor (NGF)-induced neurite growth in PC12 cells [17]. However, other group had previously reported that P2XR activation reduced the NGF-induced growth cone mobility and the filopodia length in PC12 cells by increasing the local calcium concentration at the growth cone [18]. Along this line, Iketani et al. also reported that ATP-evoked calcium transient through P2X in the growth cone of cultured chick dorsal root ganglion neurons resulted in growth cone arrest by enhancing the phosphorylation of eukaryotic elongation factor-2 (eEF2) [19]. In agreement with these studies, it was reported that extracellular ATP reduces neural cell adhesion molecule (NCAM)-induced neurite outgrowth in B35 neuroblastoma cells [20].

Considering the studies mentioned above reporting Ap_n_A-sensitive P2X receptors may play opposite effects on neurite growth, in the present work, we aim analyzing the roles that diadenosine pentaphosphate (Ap_5_A) may play in dendrite and/or axonal growth using the well-established model system of cultured hippocampal neurons [21].

## Materials and methods

### Reagents

The following reagents were used in this study: Ap_5_A (D4022), TNP-ATP (T4193), and Phenol Red (P3532), all purchased from Sigma-Aldrich St Louis, USA. NF449 (1391) and NF279 (1199) were obtained from Tocris Bioscience. Ip_5_I was synthesized as previously described [22].

### Cell culture

Hippocampal neurons were prepared as described previously [21]. Briefly, the hippocampus was dissected and dissociated from E18 mouse embryos using the Papain Dissociation System (PDS; Worthington Biochemical Corp., Lakewood, NJ, USA). For pharmacological experiments, neurons were plated at a density of 10,000 cells/cm^2^ on poly-L-lysine-coated coverslips (1 mg/mL, Sigma-Aldrich, St Louis, USA), and for cell transfection, neurons were plated at 100,000 cells/cm^2^ on coverslips or 35 mm plates coated with polylysine (10 μg/mL, Biochrom A.G., Berlin, Germany) and laminin (3 μg/mL, Sigma-Aldrich St Louis, USA). After plating, neurons were cultured for 3 days in neurobasal medium (NB; Life Technologies, Inc., Gaithersburg, MD, USA) supplemented with 1% B-27, 0.5 mM glutamine, 1 mM pyruvate, 100 U/mL penicillin, and 100 mg/mL streptomycin. To analyze the effect of P2X receptor agonists and antagonists, the compounds were added to the cultured neurons 3 h after plating, at the concentrations indicated.

HEK293T cells were maintained in DMEM (Gibco) supplemented with 10% (v/v) FCS. Cells were reseeded at 10^5^cells/cm^2^ 1 day before transfection, after which FCS was reduced to 0.5% (v/v).

### Plasmid constructs and the design of shRNAs for P2X1R

The human P2X1 full length cDNA was purchased and sequenced by Geneservice Ltd. (cDNA clone number OHu21348, IMAGE: 5,206,874; Cambridge, UK). The P2X1 cDNA was isolated from the original plasmid (pOTB7) by digestion with EcoRI and BamHI, and then subcloned into the corresponding sites of pcDNA3 for expression in mammalian cells. To construct the P2X1-GFP plasmid, P2X1 was cloned into the pd2EGFP-N1 vector (Clontech) and the ligation product was confirmed by sequencing. P2X1 receptor knockdown was achieved by RNA interference (RNAi) using a vector-based small hairpin RNAs (shRNA) approach. The shRNA target sequences were selected for P2X1 receptor according to a previously reported rational design protocol [23]. As a control, we used firefly luciferase–targeted shRNAs. The specificity of the sequence was confirmed by a BLAST analysis for human, mouse, and rat P2X1. Synthetic forward and reverse 64-nucleotide oligonucleotides (Sigma Genosys) were designed, annealed, and inserted into the BglII/HindIII sites of the pSUPER.neo.GFP vector (OligoEngine, Seattle, WA) following the manufacturer’s instructions. These constructs express 19-bp 9-nucleotide stem-loop shRNAs targeting either P2X1 or luciferase (control shRNA) mRNAs. The concomitant expression of green fluorescent protein (GFP) from this vector allowed transfected cells to be identified by fluorescence.

### Cell transfection

HEK 293 T cell transfections were performed with the pSUPERneo-GFP-derived plasmid constructs in the presence or absence of P2X1-GFP using Lipofectamine 2000 according to the manufacturer’s instructions. After 6 h, the medium was removed, and the cells were further incubated for the periods indicated in the presence of culture medium. Neuronal transfection was carried out 24 h after plating using lipofectamine 2000 (12 μL, Invitrogen), 3 μg of control shRNA Luc or shRNA P2X7R vectors plus 3 μg of P2X1-GFP. The transfection mix was removed after 2 h, and the neurons were washed and maintained for 3 DIV.

### Calcium studies: microfluorimetric analysis

Hippocampal neurons were cultured on coverslips treated with polylysine as described previously. The day after the cells were plated, neurons were washed with HBM buffer (140 mM NaCl, 5 mM KCl, 1.2 mM NaH_2_PO_4_, 1.2 mM NaHCO_3_, 1 mM MgCl_2_, 10 mM glucose and 10 mM HEPES, pH 7.4), and they were loaded with FURA-2AM solution (5 μM) for 30 min at 37 °C. This period facilitated the intracellular hydrolysis of FURA-2AM. Subsequently, the coverslips were washed again with HBM medium and mounted in a superfusion chamber on a NIKON Eclipse TE-2000 microscope. Neurons were continuously superfused at 1.2 mL/min with HBM perfusion media during functional assays. Pulses of 30 s with Ap_5_A (100 µM) were applied to neurons. Neurons were visualized using a Nikon microscope containing a × 40 S Fluor 0.5–1.3 oil lens. The wavelength of the incoming light was filtered to 340 nm and 380 nm with the aid of a monochromator (10 nm bandwidth, Optoscan monocromator, Cairin). The 12-bit images were acquired with an ORCA-ER C 47 42–98 CCD camera from Hamamatsu (Hamamatsu City, Japan) controlled by the Metafluor 6.3r6 PC software (Universal Imaging Corp., Cambridge, UK). The exposure time was 250 ms for each wavelength and the changing time was < 5 ms. The images were acquired continuously and buffered in a fast SCSI disk. The time course data represents the average light intensity in a small elliptical region within each cell. The background and autofluorescence components were subtracted at each wavelength.

### Immunofluorescence studies

Immunofluorescence was performed on hippocampal neurons cultured for 3 DIV fixed with 4% paraformaldehyde. Nonspecific binding was blocked with 1% bovine serum albumin (BSA), 5% fetal bovine serum (FBS), and 0.2% Triton X-100 in phosphate-buffered saline (PBS). The cells were then incubated with primary antibodies: polyclonal anti-GFP (1:1000), monoclonal anti-β-III-tubulin (1:1000), or polyclonal anti-P2X1 (1:50) for 1 h at room temperature. Coverslips were washed three times with 1% BSA in PBS and incubated with Alexa-Fluor-488 secondary antibody (1:400). Coverslips were mounted using FluorSave (Calbiochem, Nottingham, UK), and images were acquired using a Nikon TE-200 fluorescence microscope coupled to a Kappa DX2 camera. Analysis of axon length and ramifications were carried out using the Image J software v.1.41o. Images were processed and presented using Adobe Photoshop and Illustrator CS3.

### Western blotting

Transfected HEK 293 T cell were washed with PBS, lysed, and homogenized in a RIPA (Radio-Immunoprecipitation Assay) lysis buffer (50 mM Tris–HCl, pH 8.0, 150 mM sodium chloride, 1.0% Igepal CA-630 [NP-40], 0.5% sodium deoxycholate, and 0.1% sodium dodecyl sulphate) (Sigma-Aldrich, St Louis, USA) supplemented with Complete Protease Inhibitor Cocktail Tablets (Roche Diagnostics GmbH, Germany). Proteins were separated on 10% SDS-PAGE gels and transferred to nitrocellulose membranes (PROTRAN Nitrocellulose Transfer Membrane BA 85, Whatman, USA), saturated for 1 h at room temperature with 5% non-fat dried milk or 3% BSA in PBS, and incubated overnight at 4 °C with the following primary polyclonal antisera (and dilutions): polyclonal anti-GFP (1:1000) and the monoclonal anti-β-actin antibody (1:1000). A secondary goat anti-mouse monoclonal antibody (1:5000) or goat anti-rabbit polyclonal antiserum (1:1000) coupled to horseradish peroxidase (Dako, Glostrup, Denmark) were used to detect the primary antibodies, which were visualized by ECL (Perkin Elmer, MA, USA).

### Statistics

All experiments were repeated at least three times, and the results are presented as means ± s.e.m. Statistical differences were analyzed as indicated using the GraphPad Prism 5 software and unpaired *t*-test or one-way ANOVA test followed by Dunnett post hoc test.

## Results

### Ap_5_A decreases dendritic length and number by increasing the calcium concentration in the dendrites of cultured hippocampal neurons

**Fig. 1:**
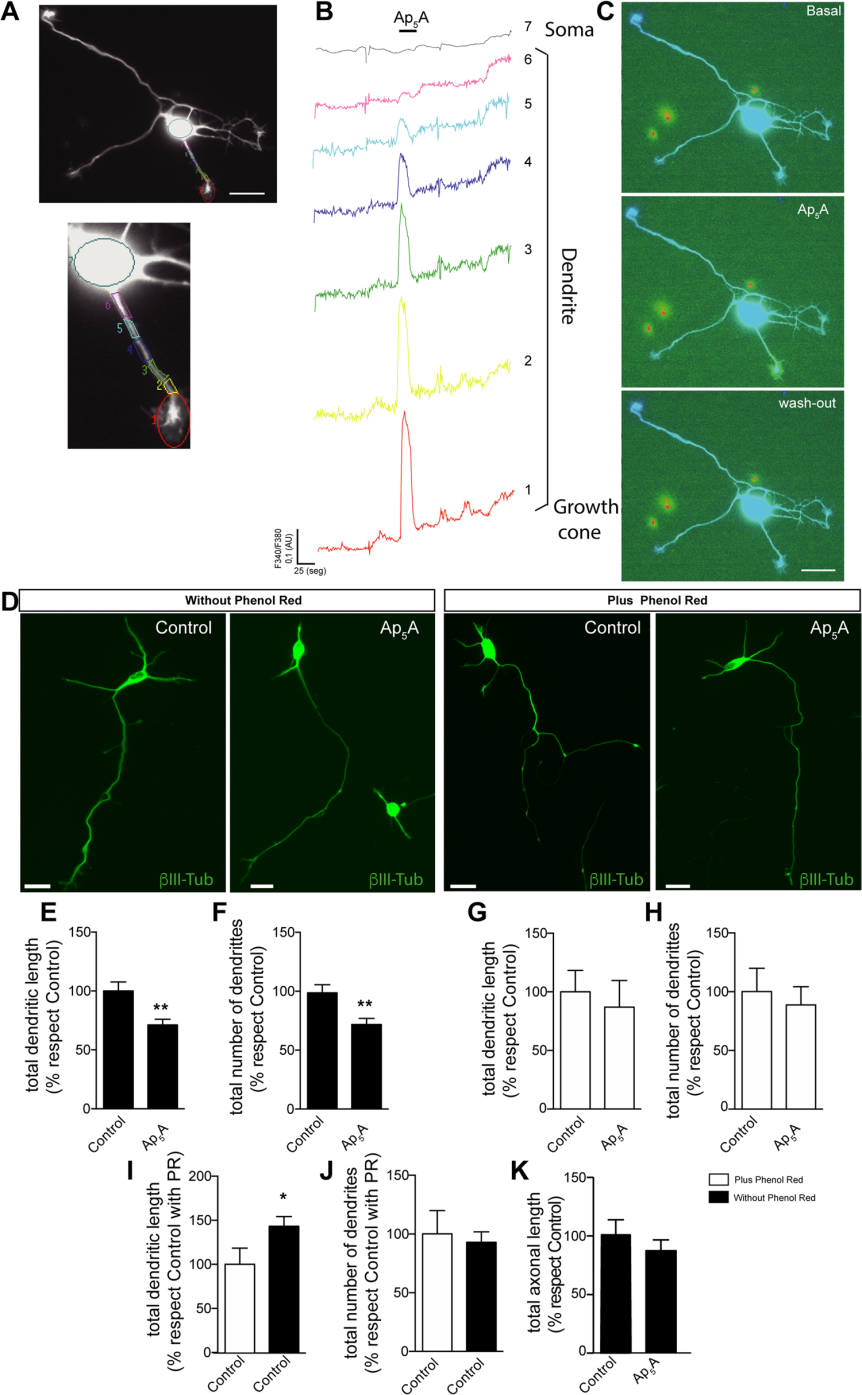
Ap_5_A induces intracellular Ca.^2+^ transients in distal dendrite regions and exerts a negative effect on dendrite growth and number. **A** Fluorescence image of hippocampal neurons loaded with Fura-2 dye. Scale bar: 25 μm. 2.5 × magnification image of selected region in A, which showed six different areas along the dendrite (from the growth cone in region 1 to the most proximal part of the dendrite to soma in region 6), and the soma (region 7) were analyzed. **B** The graphs represent the time course of Fura-2 emission as the 340 (F340) and 380 (F380) wavelength ratio. Solid bars represent the time period for 100 µM Ap_5_A assayed. AU arbitrary units. **C** Panels show representative images of changes in Fura-2 fluorescence recorded at three different times during the experiment (basal, Ap_5_A stimulation, and wash out after stimulation). Scale bar: 25 μm. **D** Hippocampal neurons cultured for 3 days in the presence or absence of 100 µM Ap_5_A and/or phenol red. Neurons were stained for β-III-tubulin to identify the neuronal morphology a. Scale bar: 25 μm. Graphs represent the mean ± s.e.m. of the dendritic length (**E**, **G**, and **I**), total number of dendrites (**F**, **H**, and **J**) and axonal length (**K**) in four different experiments (*n* = 80). Statistical differences were analyzed using a paired *t*-test (*P < 0.05, **P < 0.01 versus control)

To evaluate whether the diadenosine polyphosphates can modulate the neurite growth dynamic, we used a well-established model of cultured hippocampal neurons [21]. Given that these compounds may bind both to ionotropic and metabotropic purinergic receptors [13, 14], we first tested whether Ap_5_A could mobilize calcium in neurites. To this end, neurons were plated on coverslips and cultured to stage 3 before they were loaded with fura-2 dye (Fig. 1A). At this stage, neurite elongation is evident; in particular, the longest neurite will become in the axon, while the rest of neurites will evolve into dendrites [21]. When neurons were stimulated with a 100-µM Ap_5_A pulse, similar conditions to those already used to identify selective Ap_5_A-induced responses in isolated nerve endings [24], a local calcium increase was observed in the distal dendrite but not in the axon or axonal growth cone (Fig. 1B–C). Interestingly, the Ap_5_A-induced calcium wave in dendrites did not reach the somatodendritic compartment (Fig. 1A–C). To elucidate how the calcium wave induced by Ap_5_A impacts on neurite growth dynamic, we analyzed neuronal morphology after incubation of hippocampal neurons with 100 µM Ap_5_A. Given that phenol red (PR), a pH indicator present in standard neurobasal media (20,51 µM), is also a potent inhibitor of some P2X receptors, including homomeric P2X1, P2X2, and P2X3 in the micromolar range [25], we decided to culture hippocampal neurons in the presence or absence of this compound. After 72 h, neurons were fixed and stained with an anti-β-III tubulin antibody for morphological analysis (Fig. 1D). In the absence of PR, Ap_5_A caused a significative reduction both in the total dendrite length (28.7 ± 4.9%; Fig. 1E) and in their number (28.1 ± 5.0%; Fig. 1F) compared with PBS-treated neurons (which showed a mean of 298.3 ± 54.9µm for dendrite length and 4.9 ± 0.9 of dendrites). However, in the presence of PR, Ap_5_A did not modify neither the dendritic length nor number of dendrites (Fig. 1G and H). Interestingly, in the absence of any external stimuli, the lack of PR caused a higher dendritic length but did not modify the number of dendrites (Fig. 1I and J), nor the axonal length (Fig. 1K) of the cultured hippocampal neurons. Since these results seem to suggest that the presence of PR in the culture medium blocks the receptor sensitivity to Ap_5_A, we decided to use a Neurobasal media lacking PR for the next experiments.

### P2X1 subunit is involved in Ap_5_A-activated P2X receptor modulating dendrites length and number

**Fig. 2:**
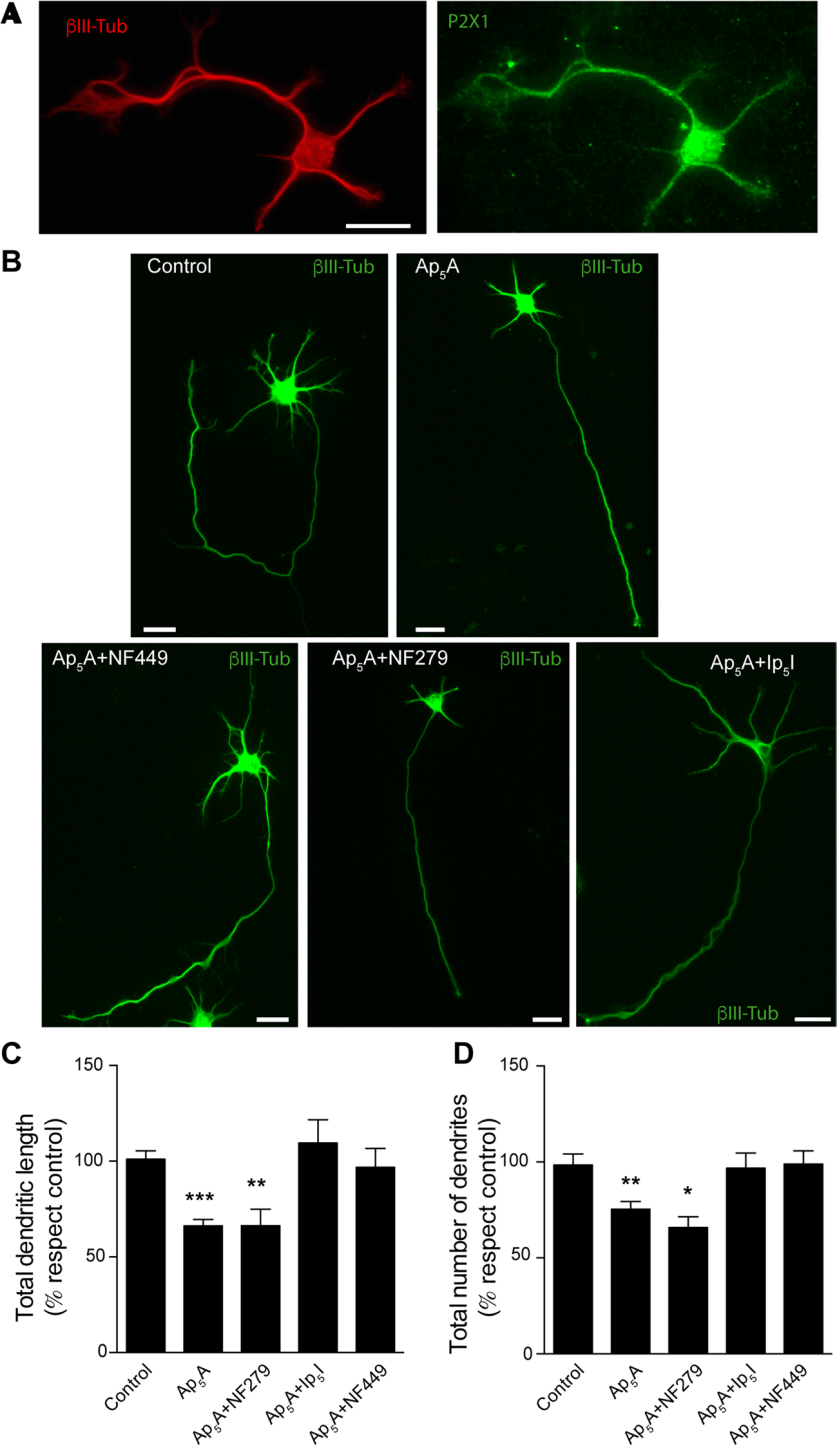
Purinergic receptors P2X1 regulate dendrite growth and number in cultured hippocampal neurons. **A** Hippocampal neurons cultured for 3 DIV stained with antibodies against β-III-tubulin (red channel) and P2X1 (green channel). Scale bar: 50 μm. **B** Hippocampal neurons cultured for 3 DIV and stimulated with 100 µM Ap_5_A in the presence or absence of the P2X1 antagonists NF279 (20 nM), Ip_5_I (10 nM), and NF449 (10 nM). Neurons were stained with an anti-β-III-tubulin antibody to observe their morphology. Scale bar: 25 μm. Quantification of dendritic length (**C**) and the number of dendrites (**D**) from the experiments shown in **B**. The data represent the mean ± s.e.m. obtained from three independent experiments each involving at least 30 neurons. Statistical differences were analyzed using a one-way ANOVA test followed by Dunnett test correction (*P < 0,05; **P < 0.01; ***P < 0.0001 versus control)

Since preliminary results suggest the involvement of P2X1, P2X2, or P2X3 subunits in the Ap_5_A-sensitive receptor present in hippocampal neurons, we initially focused on evaluating the contribution of P2X1 subunit. Double immunolabeling of hippocampal neurons for the P2X1 and β-III tubulin revealed a wide distribution of this subunit throughout the somatic-dendritic compartment as well as in axons of hippocampal neurons, including the growth cones (Fig. 2A). Afterward, to evaluate the involvement of this subunit in the above-described effects, we treated the neurons with 100 µM Ap_5_A in the presence or absence of different selective P2X1 receptor antagonists, 10 nM NF449, 20 nM NF279, and 10 nM Ip_5_I. In all cases, the antagonists were added to cultured neurons 20 min before Ap_5_A was assayed. Interestingly, whereas NF449 and Ip_5_I avoided the reduction of both the number and length of dendrites induced by Ap_5_A (Fig. 2B–D), NF279 did not prevent any of them (Fig. 2B–D). Apart from the morphological changes described above, no other evident changes were associated with any of the treatments tested.

**Fig. 3:**
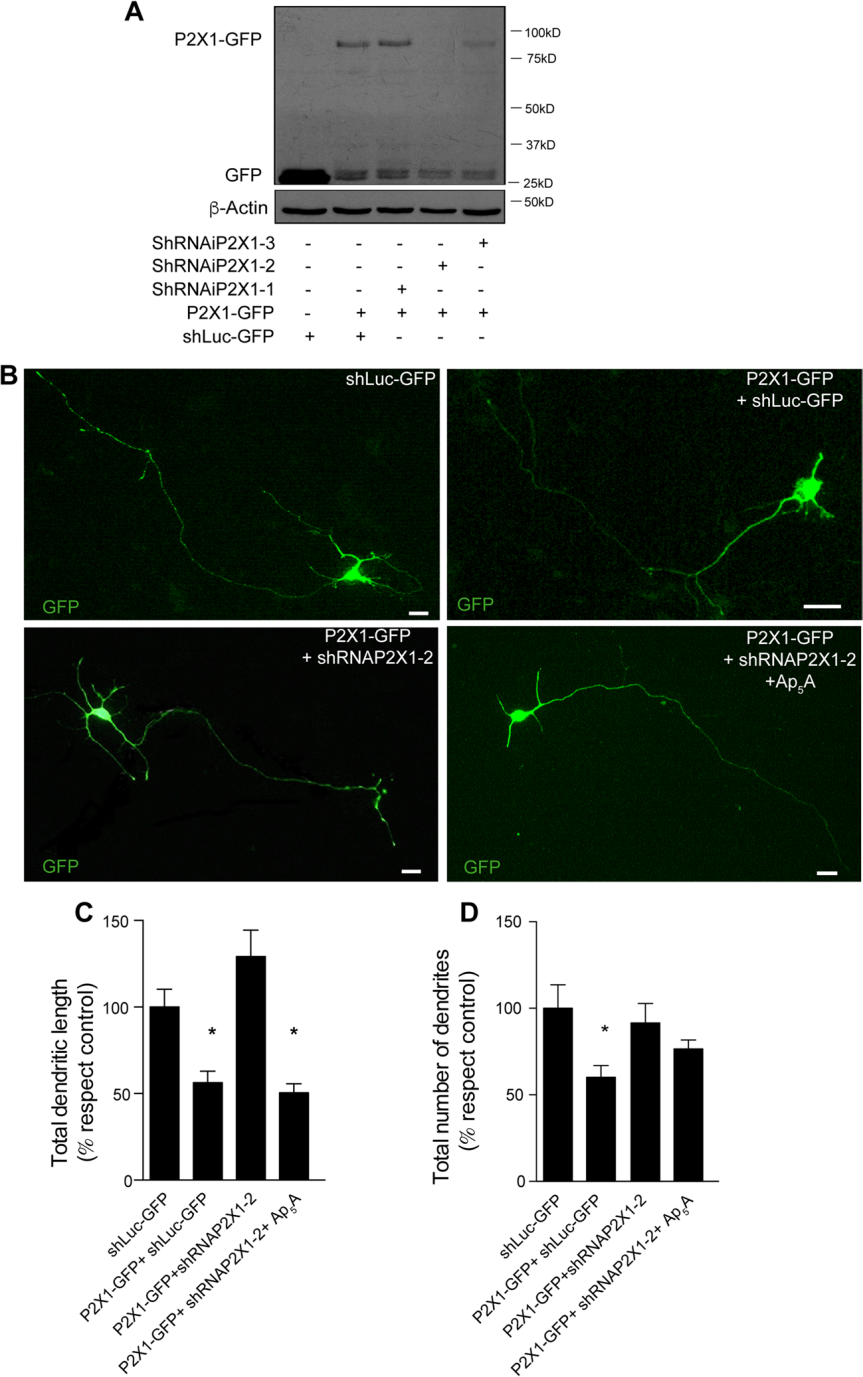
P2X1 overexpression reduces the length and number of dendrites but the P2X1R knockdown fail to revert the Ap_5_A-induced reduction of dendrite length. **A** Western blot analysis of HEK293T cells co-transfected with plasmids overexpressing P2X1-GFP plus different shRNA candidates against P2X1R. The levels of β-actin were used as a loading control. **B** Representative GFP-fluorescence images of hippocampal neurons transfected at 1 DIV with shLuc-GFP, P2X1-GFP plus shLuc-GFP, or P2X1-GFP plus shRNAP2X1-2 in the presence or absence of 100 µM Ap_5_A. Neurons were fixed, and their dendritic length and ramifications were analyzed at 4 DIV. Quantification of dendritic length (**C**) and the number of dendrites (**D**) from the experiments shown in **B**. The data represent the mean ± s.e.m. obtained from three independent experiments each involving at least 15 neurons. Scale bar: 25 µm. Statistical differences were analyzed using a one-way ANOVA test followed by Dunnett test correction (*P < 0.05 versus control)

Given the non-conclusive results reached using a pharmacological approach, we adopted a second strategy based on molecular biology approaches for overexpressing and silencing the P2X1 receptor. We designed three pSUPER.neo.GFP vectors derived shRNA strategy to target the P2X1 receptor (see “Materials and methods”). The effectiveness of this shRNA-P2X1 approach was first confirmed in the HEK293T cell line. To do that, this receptor was first expressed in HEK293T cells using a pd2EGFP-N1-P2X1 vector, which encodes for a functional P2X1 receptor fused with the GFP protein (P2X1-GFP), and these cells were also co-transfected with either the vectors expressing each one of three shRNAs for P2X1 or with an unspecific shRNA (shLuc-GFP) (Fig. 3A). The expression of exogenous P2X1-GFP was specifically abolished in cells co-transfected with shRNA-P2X1-2 (Fig. 3A). Having confirmed the efficiency and specificity of the shRNA-P2X1-2, hippocampal neurons were co-transfected with P2X1-GFP in the presence of shLuc-GFP, or shRNA-P2X1-2 vectors at 1 day in vitro (DIV), and these neurons were fixed at 4 DIV to examine the length and ramifications of their dendrites. As controls, additional sets of neurons were similarly transfected with shLuc-GFP. Like those exposed to Ap_5_A (Fig. 1D–G), neurons transfected with P2X1-GFP + shLuc-GFP had significantly shorter dendritic tree (43.7 ± 6.7%) and lower number of dendrites (39.8 ± 6.7%) than neurons transfected with a plasmid expressing shLuc-GFP alone (Fig. 3B–D). Nevertheless, neurons co-transfected with P2X1-GFP plus shRNA P2X1-2 showed a similar number of dendrites and a larger dendritic tree than shLuc-GFP transfected control neurons (Fig. 3B–D). As expected, when neurons co-transfected with P2X1-GFP plus shRNA-P2X1-2 were stimulated with 100 µM Ap_5_A, they showed a similar number of dendrites than those detected in neurons transfected with shLuc-GFP (Fig. 3 B and D). However, these neurons unexpectedly had shorter dendrites than those transfected with shLuc-GFP (39.1% ± 5.4; Fig. 3B–C). In summary, both pharmacological and molecular biology approaches suggest that the Ap_5_A-sensitive P2X receptor is a heteromeric P2X, assembled by P2X1 subunits and others.

### Possible involvement of other P2X subunits in Ap_5_A-sensitive P2X receptor

**Fig. 4:**
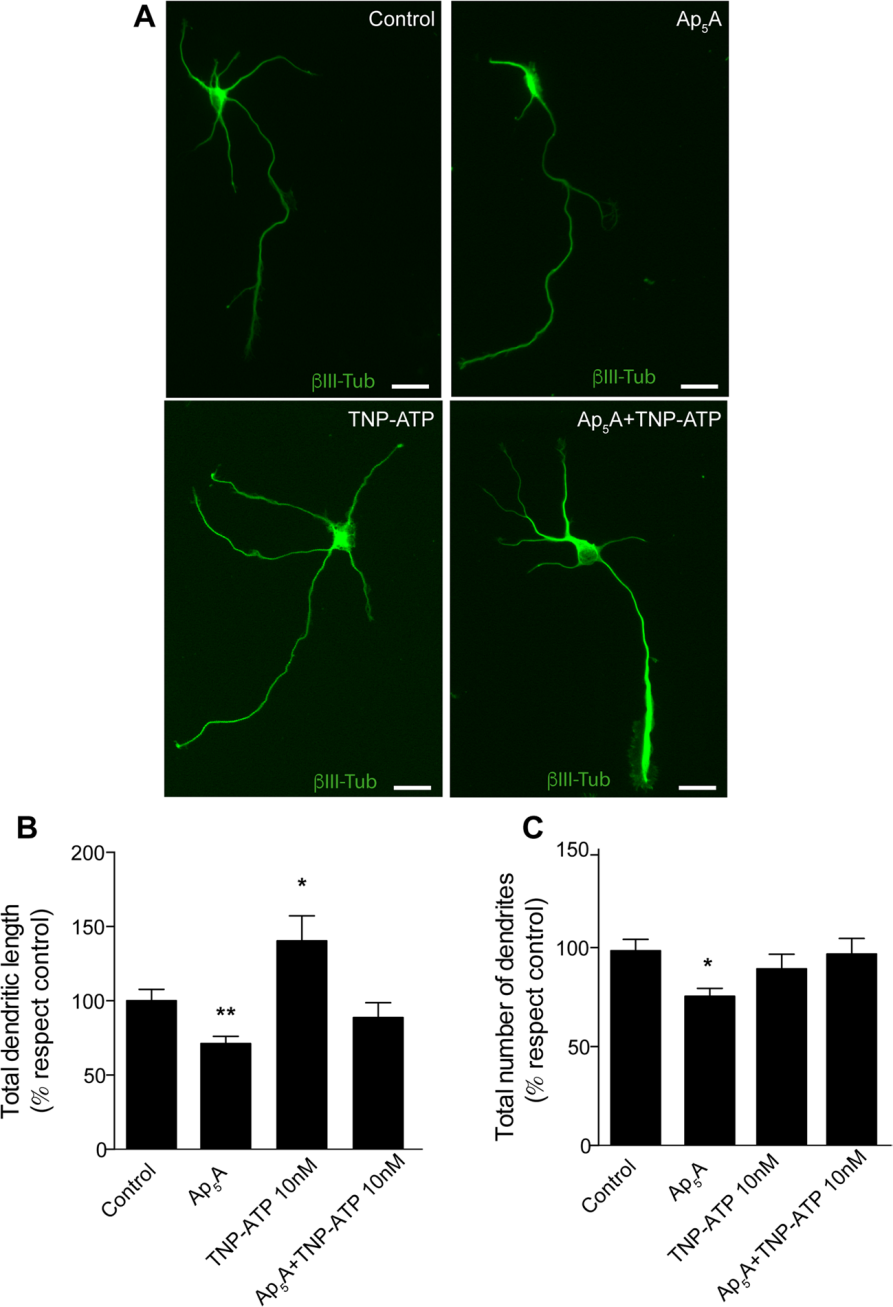
Possible implication of a heteromeric P2X receptor. **A** Hippocampal neurons were cultured for 3 DIV in the presence or absence of the P2X3 antagonist TNP-ATP (10 nM) and/or 100µM Ap_5_A. Neurons were stained with an anti-β-III-tubulin antibody to observe their morphology. Scale bar: 25 μm. Quantification of dendritic length (**B**) and the number of dendrites (**C**) from the experiments shown in **A**. The data represent the mean ± s.e.m. obtained from three independent experiments each involving at least 25 neurons. Statistical differences were analyzed using a one-way ANOVA test followed by Dunnett test correction (*P < 0,05; **P < 0.01 versus control)

To evaluate the involvement of other P2X subunits, specifically P2X3, in the Ap_5_A-induced effects on dendrites, we resorted again to pharmacological approaches using additional selective P2X receptor antagonists, such TNP-ATP [26, 27]. Results revealed that 10 nM TNP-ATP caused a significant increase in the dendritic tree length but not in the number of dendrites when tested alone. Remarkably, the pre-treatment of neurons with TNP-ATP avoided the reduction in the number and length of dendrites induced by Ap_5_A (Fig. 4A–C).

## Discussion

In the present work, we provide the first evidence indicating that diadenosine polyphosphates, specifically Ap_5_A, regulate the dendrite number and its growth in hippocampal neurons by inducing local Ca^2+^ increases in the neurite growth cone. In agreement with our results, previous works reported ATP-evoked calcium transients through P2XR in growth cones resulting in a growth arrest both of neurites [18, 19] and axons [7]. Given that we did not observe that Ap_5_A induces any local Ca^2+^ influx into the axonal growth cone nor any effect on axonal growth, we postulate that diadenosine polyphosphates have a selective effect on dendrite growth. Contrary to these results, other groups have reported that P2X activation promotes neurite outgrowth [15, 17]. This apparent contradiction might be due to the fact that external cues induce calcium influx that may module the growth cone motility by different mechanisms. In this way, it was reported that high calcium influx promotes attraction, low calcium promotes repulsion, and mid-range causes random growth [28]. Likewise, it was reported that a relatively large intracellular Ca^2+^ increase on one side of the growth cone activates calcium-calmodulin-dependent protein kinase II (CaMKII) causing a growth cone attraction towards the activated side, while a modest calcium signal activates calcineurin (CaN) results in growth cone repulsion [29]. A later study in hippocampal dendritic spines demonstrated that CaMKII is only activated when the input frequency of the calcium signal is high, whereas CaN respond to low-frequency transients [30]. Based on above-mentioned evidence and considering the Ap_5_A-induced morphological changes in dendrites, it would be expected that P2X receptor sensitive to Ap_5_A would induce local and transient calcium influx. In line with this hypothesis, we observed that Ap_5_A induces a local and modest calcium influx in the dendrite growth cone that did not reach the somatodendritic compartment. Agree with our observation, other groups have described that Ap_5_A behaves as a partial agonist of P2X1R [11]. Moreover, it has been postulated that P2X1 may form macromolecular complex with kinases and phosphatases, including those that regulate NFAT activation, such as CaN [31].

Pharmacological studies conducted with well-characterized P2X1R antagonists such as phenol red [25], NF449 [17, 32, 33], and Ip_5_I [34] suggested the involvement of homomeric P2X1R in the arrest of dendritic growth induced by Ap_5_A. Immunological studies confirmed the presence of P2X1 subunits in the dendritic growth cone. Supporting pharmacological and immunological studies, overexpression of a functional P2X1-GFP receptor reduced the length and number of dendrites in cultured hippocampal neurons, whereas the selective P2X1-GFP knockdown using shRNAs prevented the reduction of dendrite length. In line with our results, it was previously reported the presence of P2X1 in neuronal fibers of both hippocampal granular and pyramidal neurons [35]. However, the fact that the selective P2X1-GFP knockdown avoided the Ap_5_A-induced decrease of dendrites number but not the reduction of their length, and the lack of effect of some assayed selective-P2X1 antagonists, such as NF279, invite to consider the possible involvement of a heteromeric P2X1 receptor in the Ap_5_A-induced effects. In agreement with this hypothesis, we found that although the P2X1 subunit is widely distributed both in the somatodendritic compartment and the axon, only the dynamic growth of dendrites is affected by the Ap_5_A-sensitive P2X receptor. These results suggest that other P2X subunits, such as P2X2 or P2X3 subunits with which they make up functional heteromeric receptors, might be directing the expression of the Ap_5_A-sensitive heteromeric P2X1 receptor to dendrites. In accordance with this hypothesis, we found that TNP-ATP a potent P2X3 receptor antagonist [26] also avoided the Ap_5_A-induced decrease of dendrites growth and number. These results might indicate the possible involvement of a P2X1/3 receptor in the polyphosphates mediated actions. Nevertheless, since TNP-ATP can also block the P2X1 homomeric receptor but with less potency than the P2X3 [26], we cannot exclude that TNP-ATP-induced effects was through P2X1R. On the other hand, the fact that TNP-ATP promotes by itself dendrite elongation in the absence of any other stimuli reveals a tonic activation of ATP-sensitive neuronal receptors. Supporting this idea, in previous work, we found significant levels of ATP in the supernatant of the culture medium of hippocampal neurons. However, in the same work, we also described that extracellular ATP levels declined along the culture days due to increased expression of the ectonucleotidase tissue-nonspecific alkaline phosphatase (TNAP) in neurons [36]. Both findings raise two relevant questions: (i) Could the tonic activation of other ATP-sensitive receptors condition Ap_5_A-induced effects on dendritic growth? and (ii) since a previous study reported that adenosine A_2A_ receptor contributes to the radial migration of cortical projection neurons through regulating neuronal polarization and axon formation [37], could adenosine from extracellular hydrolysis of basal ATP affect the Ap_5_A-induced effects on the dendritic growth? Although our results confirm the involvement of the heteromeric P2X1 receptor in the Ap_5_A-induced effects, additional studies should be done to evaluate whether tonic activation of ATP- or adenosine-sensitive receptors might affect Ap_5_A-induced dendritic growth and branching.

Here, we provide the first evidence indicating Ap_5_A regulates the dendrite growth and number in cultured hippocampal neurons. Since we previously demonstrated that ATP via P2X7R regulates axonal growth and branching and considering extracellular breakdown of Ap_5_A by ecto-nucleotidases generate nucleotides, including ATP, the present results reveal the complex regulation that purinergic signaling exerts on the neuronal differentiation.

## Data Availability

The datasets used and/or analyzed during the current study are available from the corresponding author on reasonable request.
